# Lemborexant for the Treatment of Insomnia in Patients From China: Four Case Studies

**DOI:** 10.7759/cureus.64655

**Published:** 2024-07-16

**Authors:** Weiying Jian, Minyan Feng, Jin Li

**Affiliations:** 1 Department of Mental Health, Guangzhou United Family Hospital, Guangzhou, CHN; 2 Department of Pharmacy, Guangzhou United Family Hospital, Guangzhou, CHN

**Keywords:** general anxiety disorder-7, patient health questionnaire-9, insomnia severity index, china, insomnia, lemborexant

## Abstract

Lemborexant is a competitive antagonist of dual orexin receptors indicated for the treatment of adult patients with insomnia characterized by difficulties with sleep onset and/or sleep maintenance that occur at least three nights per week for ≥3 months, although there is adequate opportunity for sleep. There has been no published study of its efficacy and safety in treating patients from China with insomnia. In this case report, we present four adult patients from China with insomnia who were successfully treated with add-on lemborexant or switched from benzodiazepines or Z-drugs to lemborexant. None of the four patients experienced any discomforts related to lemborexant use. In conclusion, lemborexant treatment was effective and safe in treating the four patients from China with insomnia in this case report, and more studies are warranted to further assess the efficacy and safety of lemborexant in treating patients from China with insomnia.

## Introduction

Insomnia is a sleep-wake disorder characterized by difficulty in sleep initiation and/or maintenance that occurs three nights or more per week for at least three months, although there is adequate opportunity for sleep, according to the Diagnostic and Statistical Manual of Mental Disorders, Fifth Edition (DSM-5) [1,2]. It is a common disorder that negatively impacts various aspects of human life and increases social and healthcare costs [3]. The estimated prevalence of insomnia in China was 15.0% [3]. Although cognitive behavior therapy (CBT) is the first-line treatment for insomnia, pharmacotherapy becomes necessary for many patients with insomnia who find CBT ineffective or inaccessible [2]. Both gamma-aminobutyric acid (GABA) type-A receptor agonists, such as benzodiazepines and nonbenzodiazepine Z-drugs, and dual orexin receptor antagonists (DORAs), are commonly used pharmacological treatments for insomnia [1,2].

Lemborexant is a competitive DORA approved for treating adults with insomnia in the United States, Japan, Canada, Australia, and some other countries during recent years [1,4] and is currently under review by the Center for Drug Evaluation (CDE) in China. Lemborexant has a novel structure and can quickly bind to and separate from orexin receptor types 1 and 2 (OX1R and OX2R) [1,5]. Therefore, it can inhibit orexin-mediated wake drive by suppressing orexin binding to OX1R and OX2R over a short duration [1,5]. Two crucial phase 3 randomized controlled studies established the short-term and long-term efficacy and safety of lemborexant in improving sleep onset and maintenance [1,2,5,6]. There has been no published study of its efficacy and safety in treating patients from China with insomnia. Among the published randomized controlled trials (RCTs) and real-world studies of lemborexant as a treatment for insomnia, only the phase 3 RCT SUNRISE 2 enrolled four patients originally from China among its 949 patients, and no independent analysis was performed for these four patients originally from China [1,7]. As there is no data on lemborexant treatment for patients from China with insomnia, we present four adult patients from China with insomnia treated with lemborexant (5 mg or 10 mg every night (qn)) in this case report. This case report could provide the basis for future studies on this topic as well as a reference for physicians in China who wish to use lemborexant as a treatment for insomnia.

## Case presentation

All four patients were outpatients and provided written informed consent to the treatment and to participate in this case report. All four of these patients were diagnosed with insomnia according to the DSM-5 criteria. Patient-reported (subjective) outcomes were recorded at baseline and at each follow-up visit. The primary outcomes were sleep quality evaluated by the Insomnia Severity Index (ISI). Patients’ depression and anxiety were also evaluated by the Patient Health Questionnaire-9 (PHQ-9) and General Anxiety Disorder-7 (GAD-7) at baseline and after four weeks of treatment, respectively. The results are shown in Table 1.

**Table 1:** Demographics, ISI, PHQ-9 and GAD-7 scores of the four patients right before (baseline) and after four weeks of lemborexant treatment ISI: Insomnia Severity Index; GAD: general anxiety disorder; PHQ-9; Patient Health Questionnaire-9; M: male; F: female; QOL: quality of life

No.	Age, sex	Duration of insomnia	Mental health history	ISI									PHQ-9	GAD-7
					Total score	Falling asleep	Staying asleep	Waking up too early	Satisfaction	Impaired QOL	Worried/distressed	Daily functioning		
Case 1	31, F	4 months	NA	Baseline	18	4	4	1	3	2	2	2	6	4
				4 weeks	7	3	2	0	1	0	1	0	5	4
Case 2	43, M	3 years	NA	Baseline	11	4	3	0	2	1	1	0	3	1
				4 weeks	3	2	1	0	0	0	0	0	2	0
Case 3	50, M	20 years	Depression and anxiety	Baseline	22	3	3	4	4	3	3	2	5	1
				4 weeks	12	0	2	1	2	2	3	2	2	0
Case 4	62, F	1 year	GAD	Baseline	11	1	2	1	2	2	2	1	1	2
				4 weeks	5	0	1	1	1	1	0	1	0	0

Case 1

A 31-year-old unmarried female white-collar worker was diagnosed with insomnia as her sleep quality had been poor for more than four months. She had been having difficulty falling asleep (subjective sleep onset latency [sSOL] >2 hours), and she slept lightly and woke up easily, although she could fall back asleep. Self-medication with zolpidem improved her sleep initiation. The patient was concerned with potential dependence and side effects associated with long-term zolpidem use. We instructed her to stop taking zolpidem and instead take lemborexant (5 mg/qn). On her follow-up visit one month later, the patient reported that her sleep quality had been fair; that is, she could fall asleep within 15 minutes of going to bed; she occasionally woke up once during the night but could fall back asleep within 15 minutes. Her total sleep time was more than eight hours, and she did not feel drowsy, fatigued, or any other discomfort. The patient was satisfied with her sleep quality and wished to discontinue the treatment. A telephone interview one month after lemborexant discontinuance revealed that the patient still slept well, although it took a little longer for her to fall asleep (sSOL <30 minutes). She still woke up occasionally during the night, but she could fall back asleep, and her total sleep time was six to seven hours per day. Her changes in ISI (including total score and scores of individual items), PHQ-9, and GAD scores were listed in Table 1.

Case 2

A 43-year-old married man with a job in e-commerce was diagnosed with insomnia because he had been having difficulty with sleep initiation for the previous three years. His main complaint was difficulty in sleep onset (sSOL >2 hours), and his poor sleep quality affected his job performance. He became nervous and attempted to use lorazepam to treat his insomnia. The patient reported that with lorazepam, he had a fair sleep onset, and his total sleep time was >7 hours. However, he felt fatigued in the morning if he had to get up early. After the dosage of lorazepam was reduced, the patient had unsatisfactory sleep quality and felt the need to adjust his sleep medication. Therefore, lorazepam was discontinued, and he was prescribed lemborexant 5 mg qn. During the first three nights of the treatment, it took him >1 hour to fall asleep. We advised him to increase the dosage of lemborexant to 10 mg qn. During the follow-up visit one month later, the patient reported that he had good sleep, could fall asleep within 30 minutes of going to bed, had fair sleep maintenance, and that his total sleep time was seven hours. In addition, he did not feel fatigued, drowsy, or any other discomfort. His changes in ISI (including total score and scores of individual items), PHQ-9, and GAD-7 scores after four weeks of lemborexant treatment are listed in Table 1.

Case 3

A 50-year-old married man working at a public institution experiencing insomnia for 20 years, accompanied by depression and anxiety at the time, was diagnosed with insomnia at our clinic. Medical history revealed that the patient was diagnosed with insomnia, depression, and anxiety in 2002 at a local hospital, as he had been having difficulties with sleep initiation and maintenance as well as having early morning awakenings accompanied by anxiety and depression. He unilaterally discontinued the initial trazodone treatment after one month as it was ineffective, and he experienced recurrent symptoms and gradually aggravated difficulty sleeping over the following years. He was then prescribed citalopram and clonazepam at a hospital in Guangzhou in 2019. He unilaterally stopped taking citalopram in 2020 as his mood improved, and he was no longer obviously anxious or depressed, although he still had an early awakening at around 4 a.m. and could not fall back asleep. He kept taking clonazepam (1-2 mg/qn). The patient’s Epworth Sleepiness Scale (ESS) was 0 when he visited our clinic.

We instructed him to take lemborexant (5 mg/qn), and the dosage of clonazepam was reduced to 1 mg/qn. After four weeks, he did not have much difficulty with sleep onset, although he still had occasional sleep interruptions and still woke up easily. He felt fatigued during the daytime, and his ESS was 7. Treatment for the patient was then adjusted to lemborexant 10 mg/qn and clonazepam 0.5 mg/qn. After four weeks of the modified treatment, he had no difficulty falling asleep, had better sleep continuity, and was less fatigued during daytime. His ESS was 1. His ISI score continued to decrease from 12 before the modified treatment to 8, and his PHQ-9 score also decreased from 2 to 1.

We then instructed the patient to take clonazepam as needed, so that it was taken <4 times per week. His ISI (including total score and scores of individual items), PHQ-9, and GAD-7 scores before and after four weeks of emborexant treatment were described in Table 1.

Case 4

A 62-year-old retired married woman visited our clinic for a recurrence of mood instability and anxiety after a 30-year remission accompanied by severe insomnia for one month. Thirty years ago, she received a diagnosis of depression and underwent pharmacotherapy. She unilaterally discontinued the treatment after one month, and her condition remained stable over the years. However, the patient gradually became anxious and nervous, accompanied by severe insomnia at the end of 2022. The patient had difficulty with sleep initiation and maintenance, struggled to fall asleep, experienced panic during the day, and found it difficult to relax. She was diagnosed with generalized anxiety disorder and insomnia at our clinic.

The patient improved her sleep quality and experienced no obvious anxiety or depression with sertraline, quetiapine, or clonazepam, although her sleep continuity was still unsatisfactory. She was also fatigued during the day and had a memory decline. The patient was prescribed lemborexant (5 mg/qn) while maintaining sertraline and quetiapine and decreasing the dosage of clonazepam. She had stable sleep and mood and no physical discomfort. The patient discontinued clonazepam after two weeks, and her sleep remained stable. Two weeks after that, the patient stopped taking lemborexant unilaterally and continued taking sertraline and quetiapine for maintenance treatment. Her ISI, PHQ-9, and GAD-7 scores before and after lemborexant treatment were described in Table 1.

## Discussion

In this case report, we present four patients from China with insomnia whose sleep quality improved either by adding lemborexant or by switching from benzodiazepines or Z-drugs to lemborexant.

Studies have reported that lemborexant outperformed zolpidem extended-release in improving sleep initiation and maintenance [2], and a direct switch from zolpidem to lemborexant improved sleep maintenance in many patients [8]. Successful treatment of cases 1 and 2 was consistent with the findings on the efficacy of direct transition [8]. In addition to direct transition, one could also employ a taper method [9]. For patients with long-term use of 1-2 sedatives, starting lemborexant treatment while gradually tapering off the previous sedatives could also achieve good efficacy [5,9]. In cases 3 and 4, using lemborexant to replace clonazepam gradually improved the patients’ sleep quality. However, for those patients whose insomnia could not be effectively treated with long-term use of more than two sedatives, it probably would be better to treat these patients’ mental problems like depression and anxiety when treating their insomnia.

Using lemborexant to replace clonazepam gradually also reduced daytime fatigue in case 4, and this was consistent with previous findings that lemborexant could decrease daytime fatigue [5,10]. However, in case 3, the patient felt more fatigued during the day after a four-week treatment of lemborexant 5 mg/qn add-on to clonazepam at a reduced dosage of 1 mg/qn (ESS increased to 7 from 0), possibly because the lemborexant-clonazepam combination could increase daytime fatigue in some patients. However, we were able to reduce his daytime fatigue and further improve his sleep quality by modifying his regimen to lemborexant 10 mg/qn plus clonazepam 0.5 mg/qn (ESS decreased to 1).

The overall decrease in the ISI total score and the reduction in the scores for difficulty falling asleep, difficulty staying asleep, and/or problems waking up too early in our patients (Table 1) were consistent with the feedback from the four patients who reported that lemborexant treatment improved their sleep initiation and maintenance, as well as alleviating their early awakening. Improvement in these three aspects of sleep in the patients also led to their improved scores in the remaining four ISI items, all of which were consequences of low sleep quality.

Balancing efficacy with safety for sleep medications is crucial, and lemborexant has a very good safety profile [1,2,5,9]. Unlike the commonly used benzodiazepines and Z-drugs that broadly inhibit central nervous system activities and could lead to adverse events (AEs) such as memory decline, rebound insomnia, and withdrawal symptoms, lemborexant does not have an obvious next-morning residual effect and is not associated with cognitive impairment [5,9]. It does not lead to withdrawal symptoms when it is discontinued, as it does not have physical dependence and its abuse potential is low [5,9,11,12]. In cases 1 and 4, after the patients were treated with lemborexant for one month, discontinuing lemborexant did not result in rebound insomnia or withdrawal symptoms.

In our clinic, more than 100 patients with insomnia have been successfully treated with lemborexant, including initial treatment and switching to lemborexant as monotherapy or add-on treatment. Most of our patients were satisfied with the treatment. The unique aspect of lemborexant as a DORA is that it could potentially ameliorate problems in sleep architecture and help to normalize the sleep cycle and sleep architecture in patients with insomnia through its binding to both OX1R and OX2R [5,13]. This is probably why many patients felt that they were having a natural sleep when taking lemborexant without having a next-day hangover or being tipsy like when they took conventional sedatives. Consistent with previous reports [5,11,12], many patients treated with lemborexant discontinued the treatment after they achieved satisfactory sleep quality without having withdrawal symptoms or rebound insomnia. Somnolence and dreamfulness during sleep were the main complaints during the treatment, and most AEs were tolerable.

Finally, we found that lemborexant was very effective for treatment-naïve patients or newly onset insomnia without serious depression or anxiety and for patients like case 1 who wished to switch to a drug with low addictive potential. It could often achieve treatment goals in these patients within one month, and these patients could discontinue the treatment afterward without rebound insomnia. In addition, case 3 was a patient with a long history of insomnia (over 20 years), case 4 was a patient with general anxiety disorder and insomnia, and lemborexant successfully treated their insomnia, suggesting that lemborexant could potentially treat a wide range of patients with insomnia.

Unfortunately, all patients came from the outpatient clinic and were not assessed by subjective or objective outcome measures such as sleep diaries and polysomnography routinely used to record treatment efficacy more accurately. Therefore, treatment efficacy could only be estimated from ISI, GAD-7, and PHQ-9, all of which were heavily reliant on feedback from the patients during follow-ups. Properly designed retrospective and/or prospective studies using proper subjective and/or objective outcome measures are needed to further assess the efficacy and safety of lemborexant treatment.

## Conclusions

There has been no published study on using lemborexant to treat patients from China with insomnia. In this case report, we present four adult patients from China with insomnia who were successfully treated with add-on lemborexant or switched from benzodiazepines or Z-drugs to lemborexant. None of the four patients experienced any discomforts related to lemborexant use. Therefore, lemborexant treatment was effective and safe in treating these four patients from China with insomnia, and more studies are warranted to further assess the efficacy and safety of lemborexant in treating patients from China with insomnia.
